# First Pass Effect in Patients Treated With the Trevo Stent-Retriever: A TRACK Registry Study Analysis

**DOI:** 10.3389/fneur.2020.00083

**Published:** 2020-02-18

**Authors:** Maxim Mokin, Christopher T. Primiani, Alicia C. Castonguay, Raul G. Nogueira, Diogo C. Haussen, Joey D. English, Sudhakar R. Satti, Jennifer Chen, Hamed Farid, Candace Borders, Erol Veznedaroglu, Mandy J. Binning, Ajit Puri, Nirav A. Vora, Ron F. Budzik, Guilherme Dabus, Italo Linfante, Vallabh Janardhan, Amer Alshekhlee, Michael G. Abraham, Randall Edgell, Muhammad Asif Taqi, Ramy El Khoury, Aniel Q. Majjhoo, Mouhammed R. Kabbani, Michael T. Froehler, Ira Finch, Sameer A. Ansari, Roberta Novakovic, Thanh N. Nguyen, Osama O. Zaidat

**Affiliations:** ^1^Department of Neurosurgery and Brain Repair, University of South Florida, Tampa, FL, United States; ^2^Department of Neurology, University of Toledo, Toledo, OH, United States; ^3^Department of Neurology, Emory University School of Medicine, Atlanta, GA, United States; ^4^Department of Neurology, California Pacific Medical Center, San Francisco, CA, United States; ^5^Department of Neurointerventional Surgery, Christiana Care Health Center, Newark, DE, United States; ^6^Department of Radiology, Sidney Kimmel Medical College, Philadelphia, PA, United States; ^7^Department of Neurointerventional Radiology, St. Jude Medical Center, Fullerton, CA, United States; ^8^Department of Neurosurgery, Irvine School of Medicine, University of California, Irvine, Irvine, CA, United States; ^9^Department of Neurosurgery, Drexel Neurosciences Institute, Philadelphia, PA, United States; ^10^Department of Radiology, University of Massachusetts Medical School, Worcester, MA, United States; ^11^Department of Radiology, Riverside Radiology and Interventional Associates, Columbus, OH, United States; ^12^Department of Neurointerventional Surgery, Baptist Cardiac and Vascular Institute, Miami, FL, United States; ^13^Comprehensive Stroke Program and Neurointerventional, Texas Stroke Institute, Plano, TX, United States; ^14^Department of Vascular and Interventional Neurology, DePaul Stroke Center-SSM Neuroscience Institutes, St. Louis, MO, United States; ^15^Neurology and Interventional Radiology, University of Kansas Medical Center, Kansas City, KS, United States; ^16^Department of Neurology, St. Louis University, St. Louis, MO, United States; ^17^Department of Neurology and Neurosurgery, Los Robles Hospital and Medical Center, Thousand Oaks, CA, United States; ^18^Department of Neurology, Tulane University, New Orleans, LA, United States; ^19^Department of Neurology, Wayne State School of Medicine, Detroit, MI, United States; ^20^Department of Neurosurgery, Gundersen Health System, La Crosse, WI, United States; ^21^Department of Neurology, Neurosurgery, and Radiology, Vanderbilt University Medical Center, Nashville, TN, United States; ^22^Interventional Radiology, John Muir Health, Walnut Creek, CA, United States; ^23^Department of Radiology, Neurology, and Neurological Surgery, Feinberg School of Medicine, Northwestern University, Chicago, IL, United States; ^24^Department of Radiology, Neurology, and Neurotherapeutics, UT Southwestern Medical Center, Dallas, TX, United States; ^25^Department of Neurology, Neurosurgery, and Radiology, Boston Medical Center, Boston, MA, United States; ^26^Department of Endovascular Neurosurgery and Stroke, St. Vincent Mercy Medical Center, Toledo, OH, United States

**Keywords:** stroke, Ischemia—reperfusion, endovascualar treatment, thrombectomy, brain

## Abstract

**Background and Objective:** The first pass effect (FPE; achieving complete recanalization with a single thrombectomy device pass) has been shown to be associated with higher rates of good clinical outcomes in patients with acute ischemic stroke. Here, we investigate clinical and radiographic factors associated with FPE in a large U.S. post-marketing registry (TRACK, Trevo Stent-Retriever Acute Stroke).

**Methods:** We analyzed the TRACK database (multicenter registry of 634 patients from 23 centers from March 2013 through August 2015), which 609 patients were included in the final analysis. FPE was defined as a single pass/use of device, TICI 2c/3 recanalization, and no use of rescue therapy. Analysis of individual patient data from TRACK were performed to analyze clinical and radiographic characteristics associated with FPE as well-compared clinical outcomes defined as modified Rankin Scale (mRS) score at 30 and 90 days from hospital discharge to the non-FPE group.

**Results:** The rate of FPE in TRACK was 23% (140/609). There was no association between patient demographics and FPE, including age (*p* = 0.36), sex (*p* = 0.50), race (*p* = 0.50), location of occlusion (*p* = 0.26), baseline NIHSS (*p* = 0.62), or past medical history. There was no difference in the use of a balloon-guide catheter or general anesthesia (49 and 57% with FPE vs. 47 and 64%, *p* = 0.63 and *p* = 0.14, respectively). Clinical outcomes were significantly associated with FPE; 63 vs. 44% in non-FPE patients achieved mRS 0–2 at 90 days (*p* = 0.0004).

**Conclusion:** Our study showed that achieving complete recanalization with a single thrombectomy pass using the Trevo device was highly beneficial. The most common clinical factors that are used to determine eligibility for endovascular therapy, such as NIHSS severity, location of occlusion or patient age were not predictive of the ability to achieve FPE.

## Introduction

Recanalization is a very powerful predictor of a good clinical outcome in patients with acute ischemic stroke (AIS) from emergent large vessel occlusion (ELVO) who are treated with endovascular thrombectomy (ET). Analysis of angiographic recanalization in the HERMES collaboration of pooled patient-level data from seven trials (MR CLEAN, ESCAPE, REVASCAT, SWIFT PRIME, EXTEND IA, THRACE, and PISTE) showed a strong association of the benefit of endovascular therapy with the degree of reperfusion achieved during ET (1). Recently, the first-pass effect (FPE) defined as complete recanalization achieved with a single thrombectomy device pass was described as a novel metric of ET success (2). First described in the analysis of the North American Solitaire Acute Stroke (NASA) Registry database, FPE served as an independent predictor of good clinical outcome after stent retriever thrombectomy (modified Rankin Scale score 0–2 in 61% in FPE vs. 35% in non-FPE cohort; *P* = 0.013) (2, 3). The benefit of FPE on clinical outcome was subsequently confirmed by the Analysis of Revascularization in Ischemic Stroke With EmboTrap (ARISE II) study of the EmboTrap thrombectomy device (4).

Interestingly, stroke severity, patient age, location of ELVO (except for the internal carotid artery [ICA] terminus clot location), use of intravenous tPA and time of stroke symptom onset to treatment were not predictive of FPE in NASA (2). The analysis of the NASA registry did show that the use of a balloon-guide catheter (BGC) was predictive of FPE. Similarly, a separate prospective multicenter observational study of patients with anterior circulation stroke treated with ET showed that the use of BGC and stent retriever deployment maneuvers (standard vs. “push and fluff” technique) were associated with FPE, arguing that technical rather clinical variables affect procedural outcomes (5).

Thus, it remains to be determined what baseline clinical and radiographic characteristics of patients with ELVO undergoing ET can predict FPE when treated with stent-retriever thrombectomy as the primary treatment strategy. The TREVO Stent-Retriever Acute Stroke (TRACK) Registry was an investigator-initiated independent US post-marketing registry that evaluated the real-life clinical experience of the Trevo device in patients with AIS from ELVO (6). The registry demonstrated the generalizability of the randomized endovascular stroke thrombectomy clinical trials in real-world clinical practice, including patients who were treated outside of the formal American Heart Association clinical guidelines. The goal of our study was to examine the clinical and radiographic factors associated with FPE in the TRACK registry.

## Methods

The detailed description of the TRACK registry is provided in the original manuscript (6). Institutional review board approval was obtained at each site. Mercy Health St. Vincent Hospital (Toledo, Ohio, USA) served as the coordinating center for TRACK. Briefly, TRACK was an operator-adjudicated multicenter registry of 624 patients from 23 centers from March 2013 through August 2015 with the aim to evaluate the use of the Trevo device in clinical practice. The inclusion criteria included the Trevo device as the first mechanical thrombectomy treatment method to restore blood flow for ELVO and patients aged>18 years old. Mercy Health St. Vincent Hospital (Toledo, Ohio, USA) served as the coordinating center for TRACK. FPE is defined as a single pass/use of device, TICI 2c/3 recanalization, and no use of rescue therapy.

Demographic and clinical data analyzed in our study included age, sex, race, medical history, occlusion location, pre-stroke mRS, and treatment details including tPA administration, time of onset to groin puncture, general anesthesia, and use of rescue therapy. Final analysis included 609 patients compared to original TRACK registry based on available patient demographic and clinical data (*n* = 15 excluded). Angiographic recanalization was defined using a modified Thrombolysis in Cerebral Infarction (mTICI) score (7). Embolization into new territory (ENT) and embolization of distal territory (EDT) were defined as treatment-related embolization into areas outside of target downstream territory and any treatment-related embolization into the target downstream territory, respectively (7). Clinical outcomes were quantified using the National Institutes of Health Stroke Scale (NIHSS) on discharge, and modified Rankin Scale (mRS) score at 30 and 90 days from the day of hospital discharge. A good clinical outcome was defined as mRS 0–2. Symptomatic intracerebral hemorrhage (sICH) was defined as any parenchymal hematoma, subarachnoid hemorrhage, or intraventricular hemorrhage associated with worsening of the NIHSS score by ≥4 points within 24 h.

### Statistical Analysis

The TRACK registry was utilized to identify a FPE subgroup. Baseline features including demographics and clinical data, treatment details, and angiographic and clinical outcomes were compared with the non-FPE patients. Statistical analyses were performed using JMP V.13 (SAS institute, Cary, North Carolina, USA). Chi square and Fisher exact were used for categorical variables and the Student's *t*-test/*F*-test for continuous variables. For all statistical analyses, *p*-values were two-sided and *p* < 0.05 was considered statistically significant.

## Results

Six hundred and nine patients were included in the FPE vs. non-FPE subgroup analysis from the TRACK registry.

The rate of FPE in TRACK was 23% of patients (140/609). The two groups had similar baseline demographics, including age (*p* = 0.36), sex (*p* = 0.50), race (*p* = 0.50), and past medical history (Table 1). There were no differences in baseline NIHSS in the cohort in general and in a subgroup of patients with NIHSS <6 (*p* = 0.62 and *p* = 0.22, respectively). Pre-stroke mRS (*p* = 0.23), admission systolic and diastolic blood pressure (*p* = 0.58 and *p* = 0.77, respectively), location of occlusion (*p* = 0.26) and mode of hospital arrival (*p* = 0.39) were also similar. The two groups had equivalent rates of intravenous tPA administration prior to thrombectomy with the Trevo device (*p* = 0.44), and times of symptom onset to puncture including subgroups treated within first 6 and 8 h of symptom onset (*p* = 0.84 and *p* = 0.90, respectively).

**Table 1:** Baseline characteristics of patients with and without first pass effect (FPE).

	**FPE (*N* = 140)**	**Non-FPE (*N* = 469)**	***P*-value**
**Demographics and clinical data**			
Age (years), Mean ± SD, Median [IQR]	65.3 ± 15.3, 66.5 [22.5]	66.5 ± 14.5, 69 [20.5]	0.36
Age > 80 years old*, n* (%)	26 (19)	84 (18)	0.90
Female sex*, n* (%)	72 (51)	224 (48)	0.50
Race, white, *n* (%)	92/139 (66)	321/463 (69)	0.50
Hypertension, *n* (%)	103 (74)	357 (76)	0.58
Atrial fibrillation, *n* (%)	57/139 (41)	179 (38)	0.55
Diabetes mellitus, *n* (%)	38 (27)	121 (26)	0.74
Hyperlipidemia, *n* (%)	76 (54)	229 (49)	0.29
Hx of smoking, *n* (%)	28 (20)	124/466 (27)	0.12
Coronary artery disease, *n* (%)	26/107 (24)	112/374 (30)	0.28
Systolic blood pressure (SBP) on admission, Mean ± SD, Median [IQR]	145 ± 25.9, 139 [31] *n* = 139	145 ± 26.5, 145 [35.25] *n* = 466	0.58
Diastolic blood pressure (DBP) on admission, Mean ± SD, Median [IQR]	78.4 ± 17.6, 78 [21] *n* = 139	77.9 ± 19.2, 78 [24] *n* = 460	0.77
Transfer, *n* (%)	65 (46)	239/468 (51)	0.39
NIHSS ≥ 6	130/138 (94)	453 (97)	0.22
Baseline NIHSS, Mean ± SD, Median [IQR]	17.1 ± 6.9, 17 [9.25] *n* = 138	17.4 ± 6.6, 17 [9]	0.62
**Occlusion location**	*n* = 140	*n* = 468	
MCA M1, *n* (%)	72 (51)	261 (56)	0.26
MCA M2, *n* (%)	18 (13)	61 (13)	
MCA M3, *n* (%)	0 (0)	6 (1)	
ACA, *n* (%)	2 (1.4)	3 (0.6)	
ICA, *n* (%)	19 (14)	77 (16)	
BA, *n* (%)	24 (17)	39 (8)	
VA, *n* (%)	3 (2)	12 (3)	
Extracranial carotid, *n* (%)	0 (0)	1 (0.2)	
PCA, *n* (%)	1 (0.7)	3 (0.6)	
Tandem, *n* (%)	1 (0.7)	5 (1)	
**Pre-stroke mRS**	*n* = 130	*n* = 431	
mRS 0, n/N (%)	98	344	0.23
mRS 1, n/N (%)	10	43	
mRS 2, n/N (%)	11	22	
mRS 3, n/N (%)	8	12	
mRS 4, n/N (%)	2	9	
mRS 5, n/N (%)	1	1	
Pre-stroke mRS 0-1, n/ N (%)	108/130 (83)	387/431 (90)	0.044

*ACA, anterior cerebral artery; BA, basilar artery; ICA, internal carotid artery; IQR, interquartile range; NIHSS, National Institutes of Health Stroke Scale; MCA, middle cerebral artery; mRS, modified Rankin score; PCA, posterior cerebral artery; SD, standard deviation; VA, vertebral artery. Rows that have mean and median values between groups, p-values correspond to mean, SD values*.

Median symptom onset to puncture time in minutes was not significantly different between groups (268 and 285 min, *p* = 0.32, Table 2). However, mean ± SD [median] procedural times (groin puncture to final angiogram) was significantly shorter in patients with FPE vs. non-FPE patients (49.6 ± 23.7 [47] min vs. 99.7 ± 53.9 [90] min, *p* < 0.0001, respectively). There was no difference in the use of a balloon-guide catheter (49 vs. 47%; *p* = 0.63) or general anesthesia (57 vs. 64%; *p* = 0.14) with FPE vs. non-FPE, respectively.

**Table 2:** Treatment details of patients with and without first pass effect (FPE).

	**FPE (*N* = 140)**	**Non-FPE (*N* = 469)**	***P*-value**
**Treatment details**			
Intravenous tPA use, n/N (%)	66/138 (48)	241/467 (52)	0.44
TOG ≤ 6 h, n/N (%)	86/134 (64)	284/453 (63)	0.84
TOG ≤ 8 h, n/N (%)	106/134 (79)	362/453 (80)	0.90
TOG > 8 h, n/N (%)	28/134 (21)	91/453 (20)	0.90
Onset to puncture in minutes, Mean ± SD, Median [IQR]	384.4 ± 352.9, 267.5 [245.25]	358.1 ± 234.8, 285 [244]	0.32
Total procedural time (Groin puncture to final angiogram) in minutes, Mean ± SD; Median [IQR]	49.6 ± 23.7; 46.5 [26.5]	99.7 ± 53.9; 90 [69]	<0.0001
Total fluoroscopic time in minutes, Mean ± SD, Median [IQR]	18.5 ± 12.5, 14.6 [10.48]	36.3 ± 23.8, 30 [26]	<0.0001
Puncture to reperfusion in minutes, Mean ± SD, Median [IQR]	46.3 ± 27.4, 38 [25.75]	89.3 ± 51, 80 [63]	<0.0001
Use of BGC, *n* (%)	69 (49)	219 (47)	0.63
General anesthesia, *n* (%)	80 (57)	301 (64)	0.14
Use of rescue therapy, n/N (%)	N/A	128/462 (28)	–
# of passes, Mean ± SD, Median [IQR]	N/A	2.2 ± 1.2, 2 [2]	–

*BGC, balloon-guided catheter; IQR, interquartile range; TOG, time of onset to groin puncture; tPA, tissue plasminogen activator. Rows that have mean and median values between groups, p-values correspond to mean, SD values*.

**Figure 1:**
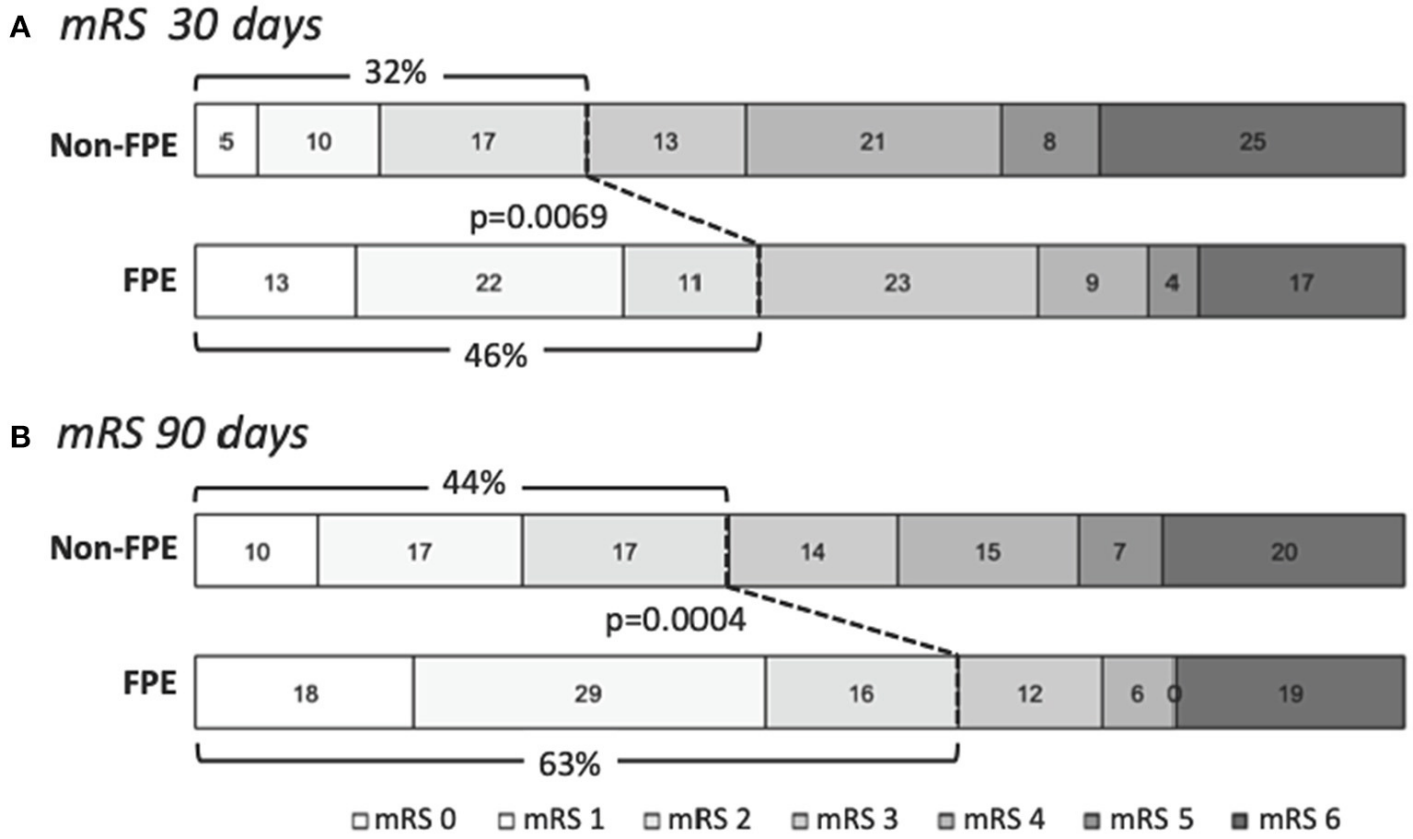
Clinical outcomes in patients who achieved first-pass effect (FPE). Clinical outcomes measured with modified Rankin scale (mRS) at **(A)** 30 days and **(B)** 90 days show significantly higher rates of good clinical outcome (mRS 0-2) in patients in whom FPE was achieved in comparison to non-FPE patients (*p* = 0.0069 and *p* = 0.0004, respectively). Numbers indicated percentages of each mRS score achieved.

Clinical outcomes were significantly associated with FPE as shown in Figure 1 and Table 3. The rates of good clinical outcome in patients with FPE were higher at 30 days (46 vs. 32% in non-FPE patients, *p* = 0.0069) and 90 days (63 vs. 44% in non-FPE patients, *p* = 0.0004). Furthermore, NIHSS at discharge was significant associated with group (FPE 9.1 ± 11.2 vs. non-FPE 12 ± 11), *p* = 0.0004. The occurrence of EDT was significantly lower in the FPE group (1/104, 1%) compared to non-FPE (110/371, 30%), *p* < 0.0001. However, no association in ENT, sICH, or death compared to FPE was present.

**Table 3:** Outcomes in patients with and without first pass effect (FPE).

**Variable**	**FPE(*N* = 138)**	**Non-FPE(*N* = 459)**	***P*-value**
**Clinical outcomes**			
mRS 0–2 at discharge, n/N(%)	38/100 (38)	83/338 (25)	0.0719
mRS 0–2 at 30 days, n/N(%)	25/54 (46)	53/165 (32)	0.0107
mRS 0–2 at 90 days, n/N(%)	77/122 (63)	177/398 (44)	0.0004
NIHSS at discharge, Mean ± SD	9.1 ± 11.2	12 ± 11	0.0004
NIHSS at 90 days, Mean ± SD	14.4 ± 18.5	19.2 ± 18.7	0.071
Death, n/N (%)	23/122 (19)	81/398 (20)	0.80
**Angiographic outcomes**			
ENT, n/N (%)	4/95 (4)	16/334 (5)	0.99
EDT in same territory, n/N (%)	1/104 (1)	110/371 (30)	<0.0001
sICH, n (%)	9 (7)	35 (8)	0.85

*EDTL, embolization in distal territory; ENT, embolization in new territory; IQR, interquartile range; NIHSS, National Institutes of Health Stroke Scale; mRS, modified Rankin score; sICH, symptomatic intracerebral hemorrhage. Rows that have mean and median values between groups, p-values correspond to mean, SD values*.

## Discussion

There is increasing evidence that achieving complete recanalization with a single thrombectomy device pass in patients with AIS from ELVO is highly beneficial. In our series of patients treated with Trevo thrombectomy device as the primary treatment strategy, 63% of patients with FPE achieved a favorable clinical outcome at 3 months, in comparison to 44% of patients without FPE. This effect translates into a 43% relative difference in achieving a good clinical outcome with thrombectomy. While FPE is strongly associated with improved clinical outcomes, our findings indicate the limitations of current thrombectomy devices; only 23% of patients treated with Trevo as the first line treatment achieved FPE. Comparable findings were demonstrated in earlier studies, although with some variations. For example, NASA analysis showed similar rates of FPE achieved with Solitaire (only 25% of cases) (2). In the ARISE II study, successful reperfusion within the first pass achieved with the EmboTrap stent-retriever device was reported 52% (4). Using the mTICI 2c/3 definition of FPE, the rate of reperfusion with EmboTrap was 40%. Furthermore, in ARISE II, thrombectomy was limited to patients presenting with 8 h of symptom onset whereas in NASA and TRACK patients were treated within a longer time window, including wake-up strokes (8). The current and prior studies strongly advocated toward further innovation in thrombectomy devices, since more than half of patients treated with stent retrievers currently do not achieve FPE.

Interestingly, our study showed that the most common clinical factors that are used to determine eligibility for endovascular therapy, such as NIHSS severity, location of ELVO, symptom onset to treatment or patient age were not predictive of the ability to achieve FPE. Likewise, in NASA most clinical and demographic factors such as stroke severity or the use of IV tPA did not influence the success of FPE with an exception of the presence of ICA occlusion, which was a negative independent predictor of FPE (2).

Factors associated with thrombectomy failure are likely multiple, including clot composition and burden, and anatomical features such as increased vessel tortuosity, according to prior investigation of factors associated with stent retriever thrombectomy failure with patients with ELVO (9–11). Unfortunately, the TRACK registry did not collect information about clot characteristics such as clot density or additional technical details, such as the length of the Trevo device to determine how these factors may influence FPE success. For example, in a single center analysis of 420 patients with ICA and MCA M1/2 occlusions treated with Trevo and Solitaire, the use of longer stent retrievers was found to be an independent predictor of FPE (12). Similar outcomes were observed in a multi-center prospective registry of patients treated with the Solitaire stent retriever (STRATIS) (13).

An unexpected finding in our study was the lack of association between the use of BGC and FPE (49% of cases with FPE and 47% of non-FPE cases utilized BGC in TRACK). By contrast in NASA, BGC use was nearly twice as common in patients who achieved FPE (64 vs. 35% in non-FPE patients) and its positive effect was confirmed by a multivariate logistic regression analysis (2). One possible explanation is that the use conjunct aspiration with Trevo (14), may have influenced the effect of BGC. Because of the limited data on the use of aspiration in conjunction with Trevo captured in our registry (the use of that technique with old generation 054 Penumbra catheter was just being introduce into clinical practice when TRACK was conducted), we were not able to perform reliable statistical analysis to examine such association. Further investigation regarding how BGC and continuous aspiration affect FPE in patients with stent-retriever thrombectomy is warranted.

The strengths of this study include the presented results were obtained based on an operator-adjudicated analysis of angiographic procedures, follow-up imaging studies, and the data were collected in a prospective manner; however, our study has limitations. The use of a Registry from 2013 to 2015 has inherent selection bias that may not be applicable to patients treated with most recent generation of thrombectomy devices. The information on important clot characteristics that may influence FPE such as clot burden score, clot length and the presence of hyperdense sign were not available. This information can be collected by the neurointerventionalist from baseline imaging, such as CT and CTA prior to beginning the thrombectomy procedure and may guide how the procedure is performed in order to achieve FPE. Several types of Trevo devices were used in TRACK, the operators' preference for such devices were not detailed, and the database did not include the information on device length/diameter used by each operator.

## Conclusions

Our study showed that achieving complete recanalization with a single thrombectomy device pass using the Trevo device in patients with AIS from ELVO is highly beneficial. However, the finding that only 23% of patients treated with Trevo as the first line treatment achieved FPE strongly advocated toward further innovation in thrombectomy devices. The most common clinical factors that are used to determine eligibility for endovascular therapy, such as NIHSS severity, location of ELVO or patient age were not predictive of the ability to achieve FPE. Further research is needed to determine how clot morphology and treatment details, including the use of BGC and conjunct aspiration, affect FPE rates.

## Data Availability Statement

The datasets generated for this study are available on request to the Principal Investigator of the TREVO Stent-Retriever Acute Stroke (TRACK) multicenter Registry.

## Ethics Statement

Ethics approval was provided by Mercy Health St. Vincent Hospital institutional review board and the ethics committee at each participating institution. The detailed description of the TRACK registry is provided in the original manuscript (6).

## Author Contributions

MM, CP, and OZ contributed to the data analysis and writing of manuscript. All authors participated in the design, conception, data gathering, editing, suggestions, and feedback of the final manuscript.

### Conflict of Interest

MM: C; Canon Medical, Cerebrotech, Imperative care, Nogueira-Stryker Neurovascular (Trevo-2 Trial Principal Investigator; DAWN Trial Principal Investigator, TREVO Registry Steering Committee), Medtronic (SWIFT Trial Steering Committee; SWIFT-Prime Trial Steering Committee; STAR Trial Angiographic Core Lab), Penumbra (3D Separator Trial Executive Committee), Neuravi (ARISE-2 Steering Committee), Genentech (Physician Advisory Board), Allm Inc. (Physician Advisory Board). OZ: C; overall PI for TRACK, Arise II, Co-PI Therapy Trial, Steering committee STRATIS registry. The remaining authors declare that the research was conducted in the absence of any commercial or financial relationships that could be construed as a potential conflict of interest. The reviewer JS declared a shared affiliation, with no collaboration, with one of the authors, VJ, to the handling editor at the time of review.
